# Short-Term Effects of a Respiratory Telerehabilitation Program in Confined COVID-19 Patients in the Acute Phase: A Pilot Study

**DOI:** 10.3390/ijerph18147511

**Published:** 2021-07-14

**Authors:** Juan Jose Gonzalez-Gerez, Manuel Saavedra-Hernandez, Ernesto Anarte-Lazo, Carlos Bernal-Utrera, Manuel Perez-Ale, Cleofas Rodriguez-Blanco

**Affiliations:** 1 Department Nursing, Physiotherapy and Medicine, Faculty of Almeria, University of Almeria, 04120 Almeria, Spain; juanjo@fisiosurid.es (J.J.G.-G.); clinicasaavedra@yahoo.es (M.S.-H.); 2 Doctoral Program in Health Sciences, University of Seville, 41009 Seville, Spain; 3 Physiotherapy Department, Faculty of Nursing, Physiotherapy and Podiatry, University of Seville, 41009 Seville, Spain; cbutrera@us.es (C.B.-U.); cleofas@us.es (C.R.-B.); 4 Spanish Army, Health Support in the Naval Base of Rota, 11520 Cadiz, Spain; manuel.manuelale@gmail.com

**Keywords:** COVID-19, physical therapy specialty, telerehabilitation, breathing exercises

## Abstract

The COVID-19 pandemic has caused distress for healthcare providers due to the respiratory problems it causes, among others. In this situation, rehabilitation of the respiratory system has been suggested and implemented in different COVID-19 patients. This study evaluated the feasibility and effectiveness of a novel program based on breathing exercises through telerehabilitation tools in COVID-19 patients with mild to moderate symptomatology in the acute stage. Forty subjects were randomized in an experimental group, based on pulmonary rehabilitation, and in a control group, of which the subjects did not perform physical activity. Thirty-eight subjects, with nineteen in each group, completed the one-week intervention. We performed measurements using the Six-Minute Walk Test, Multidimensional Dyspnoea-12, Thirty-Second Sit-To-Stand Test, and Borg Scale. Both groups were comparable at baseline. Significant differences were found for all of the outcome measures in favour of the experimental group. Ninety percent adherence was found in our program. A one-week telerehabilitation program based on respiratory exercises is effective, safe, and feasible in COVID-19 patients with mild to moderate symptomatology in the acute stage.

## 1. Introduction

The coronavirus disease 2019 (COVID-19) pandemic has led governments all over the world to adopt severe measures, including limiting interaction and imposing social distancing, to reduce spread of the virus [1]; one of the first decisions was to isolate people infected with COVID-19 exhibiting mild to moderate symptomatology for 14 days. Although necessary, this decision has becomes a barrier to providing healthcare to these patients since face-to-face contact is not possible [2] and these subjects require careful attention.

In these cases, the most common symptomatology includes fever, cough, dyspnoea, myalgia, and fatigue, among others [3]. In addition, home isolation could reduce physical activity levels and, therefore, could lead to notable deconditioning [4]; this, combined with possible sequelae due to COVID-19 [5], makes it essential to manage isolated patients properly to offer the best possible treatment for them and as soon as possible.

COVID-19 infections have been associated with lung injury and, therefore, with respiratory problems [6]. Thus, respiratory rehabilitation becomes an attractive strategy in the management of these patients, since it has been observed that pulmonary rehabilitation is effective at improving respiratory function (and, therefore, functional capacities) in different respiratory disorders, such as chronic obstructive pulmonary disease (COPD) or asthma, among others [7,8,9]. Indeed, it has been argued that rehabilitation programs that aim to improve the function of the respiratory system should be included in the management of patients with COVID-19 [10,11,12]. However, to our knowledge, only one randomized controlled trial studied the effects of respiratory rehabilitation in people infected with COVID-19, and they were elderly subjects [13].

Therefore, we find it necessary to develop a randomized, controlled trial to assess the effectiveness of breathing exercises in isolated patients with mild to moderate COVID-19. However, how could we interact with isolated patients? In that situation, telerehabilitation has become an exciting option to manage these patients. It has been recognized as an essential component of respiratory exercises, as it allows access to patients who would benefit from rehabilitation while minimizing human-to-human contact [14]. Case series and proposals have been published regarding telerehabilitation aimed at improving the respiratory system in COVID-19 patients [15,16].

Thus, we conducted a pilot randomized controlled trial to analyse the feasibility and safety of breathing exercises through telerehabilitation devices in the acute phase of COVID-19; to evaluate the financial, technical, administrative, or logistic feasibility of a full-scale study, including issues of data collection and protocol adherence; and to obtain values to calculate the sample size of a full-scale study.

## 2. Materials and Methods

### 2.1. Trial Design

The pilot trial design was a randomized, controlled, parallel, double-blind, two-arm clinical trial of the treatment. Trial registration: ACTRN12621000309886, Australian New Zealand Clinical Trials Registry. CONSORT 2010 checklist for pilot or feasibility trial is available in Supplementary Materials File S1.

### 2.2. Sample Selection

Patients were recruited through an informative text message transmitted on social networks (WhatsApp, Facebook, Instagram, Twitter, and LinkedIn), TV channels such as Deutsche Welle, radio programs, and newspapers, all of them through interviews with members of the research group in these media. They were contacted by a general message that informed them about the possibility of participating in a physiotherapy study; details about who were interested are reported later in greater detail. The recruitment was in Spain. Therefore, any patient and resident of Spain could participate in this research if he/she was a positive case diagnosed through PCR tests (polymerase chain reaction) and the antigen test, classified by each region’s epidemiology services. In terms of privacy, to start the study, the patient had to sign informed consent on the web: www.fisiosurid.com/covid-19/ accessed on 19 October 2020. Later, they were selected according to the listed eligibility criteria. The study took place at the chosen patients’ homes; evaluators carried out all measurements on the first and seventh days. All sizes were instructed and telematically controlled by the study evaluator, who provided the patients with the necessary assessment materials described below (in the Outcome Measurements section).

We excluded patients who required hospital care. The criteria were based on those published by the Spanish Society of Family and Community Medicine (SEMFYC) and can be reviewed in the Exclusion Criteria section [17].

### 2.3. Inclusion Criteria

The following were the inclusion criteria:

- Aged 18–75 years.
- Patients with positive polymerase chain reaction (PCR) test and/or antigen test results in the last forty days were in home confinement. We amplified the period to 40 days due to delays both in the performance and in the reception of the test results caused by the collapse in the Spanish Health System.

### 2.4. Exclusion Criteria

The following were the exclusion criteria, based on self-reported information provided by the subjects: patients with chronic lung conditions, chronic kidney disease, or chronic neurological disorders; patients who were suffering from hypertension and cardiovascular conditions without medical treatment; patients affected by grade III osteoporosis, by the acute phase of rheumatologic diseases, or by the acute phase of disc abnormalities; patients who had respiratory conditions in the last 12 months, who had recent musculoskeletal disorders, who were not fully recovered from their injuries, who received physical therapy treatment in the previous three months, and who were affected by chronic mental and/or psychological disturbances; red flags for severe conditions (night pain, painful muscle spasm, loss of involuntary weight, and symptom mismatch); and patients classified as moderate/severe cases based on the Spanish Society of Family and Community Medicine (SEMFYC) [17].

### 2.5. Interventions

Patients performed therapeutic exercises or sedentary activities (depending on randomized allocation to the study groups) that were exclusively assigned and could not combine these activities with other physical therapy or sports physical activity. Any interference in the treatment was grounds for exclusion. Participants were asked during daily contacts if they had carried out any action that can be considered meddling in the treatment. If participants were required to combine the intervention with medications, it was registered. Exercise monitoring was developed through telerehabilitation tools, emerging technology through which medical rehabilitation care can be provided at a distance. Patients were encouraged ultimately to carry out treatment and follow up through videoconferences that enabled them to improve their health status through their efforts and to reduce the rate of loss to follow-ups and dropouts.

#### 2.5.1. Group 1: Breathing Exercise Group

The Breathing Exercise Program was based on a proposal composed by 10 exercises. These exercises were a modified version of breathing exercises already studied in the literature [18,19]. The active cycle of breathing techniques uses an alternate depth of breathing to move mucus from small airways at the bottom of the lungs to more significant airways, where they can be cleaned more easily by coughing. The exercises are available at https://www.fisiosurid.com/exercises-covid-19/ accessed on 19 October 2020. They were carried out once a day for seven days at the patient’s home; depending on the score obtained on the Borg evaluation scale (BS), patients performed 4 (BS 7–10) (anticipated to take 10 min), 8 (BS 5–7) (expected to take 20 min), or 12 (BS < 5) repetitions per exercises a day (anticipated to take 30 min). The exercise program was reinforced by a physical therapist at least two times (if the patient did not require further attention) through telematic control by videoconference with each patient. Additionally, patients received a text message daily, asking about the exercises and as a method of follow-up and adherence.

#### 2.5.2. Group 2: Control Group

The patients in this CG underwent two assessments on days 1 and 7. These assessments were carried out by a physiotherapist who was unaware of the group to which the patient belonged. Once the data from the different evaluations were obtained, the patients were taught group 1.

#### 2.5.3. Procedure for Adverse Effects

Participants were assessed on the first and the seventh days through a phone call with a member of the study team who asked for possible adverse events. This follow-up was performed through a checklist translated and adapted from “Criteria for clinical evaluation during telephone follow-up of home care” published by SEMFYC [17].

#### 2.5.4. Outcome Measures

The data were collected by personnel related to the research group who had previously been instructed about the procedures but did not know the group to which the patients belonged; the information sent by the patients was stored and classified; and the evaluators transferred the numerical values to an excel table. The excel tables are encrypted, and only the evaluators and the lead researchers had access to it. This information was updated through a secure cloud. All outcomes were assessed through video calls on the first and the seventh days.

- Six-Minute Walk Test (6MWT) [20]. The patient’s smartphone recorded the number of steps through the app “StepsApp”. This test can determine the functional state correctly. This measure has already been evaluated through the smartphone and is considered a valid tool [21].
- Multidimensional Dyspnoea-12 (MD12). We used the already validated Spanish version of this test, a valid and reliable instrument to study the multidimensional nature of dyspnoea [22].
- Thirty-Second Sit-To-Stand Test (30STST) [23,24]. This test has been demonstrated to be a valid and reliable tool to assess the peripheral muscle performance of lower limbs.
- Borg Scale (BS). The modified version of the Borg Scale of perceived effort (0–10) [25,26] measures the entire range of activities that the individual perceives when exercising. This scale gives criteria to make adjustments to the intensity of exercise, that is, to the workload, thus forecasting and dictating the different exercise powers in sports and medical rehabilitation. Patients completed the BS at the end of the 30STST [27].

#### 2.5.5. Randomization

Patients were divided into two groups using balanced randomization, carried out with free software (http://www.randomized.org/ accessed on 10 June 2021). The principal investigator and auditor only performed the randomization sequence. No participant in the study had access to the randomization sequence, which was hidden and saved, to guarantee correct randomization with security.

#### 2.5.6. Blinding

The evaluators and patients in the study were blinded during the entire process. The evaluator was unaware of the study objectives and the randomized distribution of patients to study groups, and he did not have access to the randomization sequence. Meanwhile, although blinding for patients could not be achieved, subjects were unaware of the other treatment modalities. They did not know if they belonged to the intervention or control groups.

### 2.6. Statistical Analysis

We carried out the statistical analysis through a descriptive analysis of the data before the intervention, as a baseline, applying the Shapiro–Wilk normality test for the quantitative variables. The within-group analysis was performed based on Shapiro–Wilk *p*-values previously obtained by applying the paired t-test or Wilcoxon Z-test. The effect sizes were analysed through the R-square coefficient (R^2^), considering effect sizes lower than 0.01 as small and higher than 0.06 as medium. In contrast, an effect size higher than 0.14 is considered large. The statistical analysis was conducted at the 95% confidence level. A *p*-value of less than 0.05 was considered statistically significant in all analyses. Statistical analysis was carried out using SPSS v.26.0 (IBM, Armonk, NY, USA).

## 3. Results

**Figure 1:**
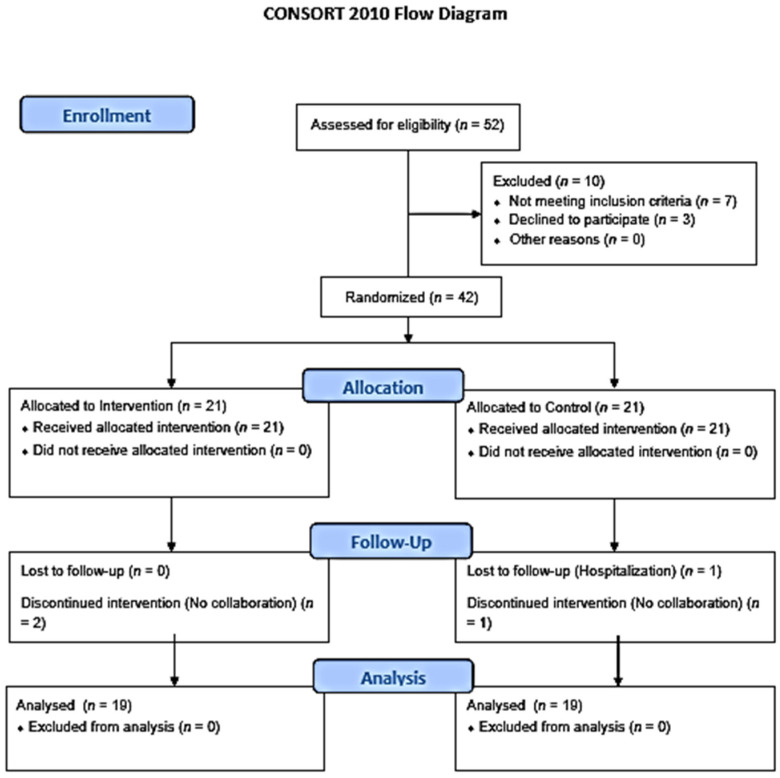
CONSORT flow diagram.

Thirty-eight patients (*n* = 38) completed the 7-day intervention and were included in the analysis. A CONSORT flow diagram can be seen in Figure 1. From patients excluded after randomization, one was due to hospitalization while three of them were excluded due to a lack of collaboration.

Both groups, the breathing exercise group (BEG) (*n* = 19; 40.79 ± 9.84 years old) and the control group (CG) (*n* = 19; 40.32 ± 12.53 years old), were comparable at baseline. We applied a normality analysis of the pre-intervention data using the Shapiro–Wilk test to analyse the distribution of the data. Table 1 summarizes the pre-intervention data for the groups.

The 7-day intervention of exercises resulted in a statistically significant improvement within groups and between groups (*p* < 0.05) (Table 2 and Table 3) in the BEG but not in the CG (*p* > 0.05). Regarding the BEG, we observed within-group differences (*p* < 0.001) in all of the variables studied. We did not observe intragroup differences in the variables of the CG (*p* > 0.05). The analysis performed for the effect size shows (Table 3) large sizes after treatment in the experimental group (r^2^ > 0.14) for all variables.

## 4. Discussion

Our study is related to the pathological condition of difficult healthcare access due to the isolation of patients with mild to moderate symptomatology who do not require hospital admission. This circumstance may make treatment of these patients less accurate, and patients may feel unprotected.

Our project implemented a new telerehabilitation program, following other rehabilitation areas such as neurologic, cardiac, or musculoskeletal, and has been driven by these exceptional circumstances generated by the pandemic [28,29,30]. Telerehabilitation in COVID-19 patients, which has been proposed in case series [16,31], presents both possible advantages and risks in large sample sizes, such as those planned in our clinical trial.

Our pilot study obtained a 90% adherence; however, 10% of the sample size did not collaborate with the study intervention concerning adherence. We understand that a 10% loss of the sample size in follow-up is acceptable and generates confidence in the implemented methods. This aspect was essential and worrying because of the type of intervention through telerehabilitation tools.

The results obtained in our study showed that subjects belonging to the experimental group achieved statistically significant changes compared to the control group in all of the variables studied and with size effects much higher than those generated in the control group, showing us the clinical relevance of the intervention.

Since the beginning of the pandemic, rehabilitation has been suggested as a management strategy of the disease [32,33] and its sequelae [34]. However, not many clinical trials have been published concerning the effectiveness of this intervention. To our knowledge, only a randomized controlled trial in elderly subjects has been published [35]. We have not found any study implementing the acute stage of these patients, so our findings may become a reference in the management of COVID-19. Hospitalized patients with severe symptomatology have been studied, benefits have been found after the implementation of respiratory training, and positive physiological parameters in blood analytics have been reported [35]. However, this information is difficult to obtain in a study such as the one we performed. It has been suggested that COVID-19 infection promotes injuries in type I and type II pneumocytes and in lung endothelial lesions, with the subsequent additional secretion of protein-rich exudate in the alveolar space and intravascular coagulation in lung vessels, which leads to a reduction in surfactant and gas exchange [36]. Therefore, the main reason for patient improvement following breathing exercises could be related to the improvement in gas exchange and the stimulation of respiratory musculature, which could lead to improvements in cardiopulmonary function and physical function reduction due to COVID-19, as already suggested [37].

We studied acute effects, and thus, long-term effects cannot be evaluated based on the results in the study, which becomes a significant limitation. In addition, a larger sample size is necessary to confirm these findings. Moreover, placebo effects could also be involved in the changes observed in our pilot study, so future research should address this limitation. However, we found that our intervention is feasible, safe, and consistent with our aims, so a more extensive study and future research are required to achieve definitive conclusions.

## 5. Conclusions

Breathing exercises through telerehabilitation appeared to provide a promising strategy for improving outcomes related to physical condition, dyspnoea, and perceived effort among people exhibiting mild to moderate COVID-19 symptoms in the acute stage, indicating clinical benefits, adherence, and safety to the program.

## Figures and Tables

**Table 1:** Descriptive pre-intervention data.

Variables	Group		Z
	*Intervention*	*Control*	*p*
Age (i)	40.79 ± 9.84	40.32 ± 12.53	0.056 ^a^
Gender (ii) *(male, female; %)*	52.6 (10/19); 47.4 (9/19)	57.9 (11/19); 42.1 (8/19)	0.744 ^c^
BS (i)	41.95 ± 4.03	48.17 ± 3.48	0.082 ^a^
MD12 (i)	12.26 ± 5.92	9.74 ± 7.26	0.054 ^a^
30STST (i)	12.68 ± 5.33	11.42 ± 3.06	0.076 ^a^
6MWT (i)	374.72 ± 151.59	393.00 ± 124.60	0.042 ^b^

Z: Shapiro–Wilk Normality Test; *p*: Statistical Significance; ^a^: T Student; ^b^: U Mann–Whitney; ^c^: Chi-Square; (i) data are expressed as means ± standard deviation; (ii) data are expressed as percentage (partial/total). BS: Borg Scale; MD12: Multidimensional Dyspnoea-12 questionnaire; 6MWT: Six-Minute Walking Test; 30STST: 30-Second Sit-to-Stand Test.

**Table 2:** Within-Group Differences.

Variables	Intervention	Valor *p*	Control	Valor *p*
BS				
Pre	5.58 ± 2.32		4.58 ± 1.89	0.292
Post	2.95 ± 1.27	<0.001 *	4.26 ± 1.85	
MD12				
Pre	12.26 ± 5.92		9.74 ± 7.26	0.822
Post	5.89 ± 3.48	<0.001 *	9.79 ± 7.47	
30STST				
Pre	12.68 ± 5.33		11.42 ± 3.06	
Post	14.00 ± 5.47	0.001 *	11.11 ± 3.78	0.629
6MWT				
Pre	374.72 ± 151.59		393.00 ± 124.60	
Post	487.58 ± 133.36	0.006 *	399.00 ± 126.07	0.144

BS: Borg Scale; MD12: Multidimensional Dyspnoea-12 questionnaire; 6MWT: Six-Minute Walking Test; 30STST: 30-Second Sit-to-Stand Test; within-group *p*-values come from paired *t*-test/Z Wilcoxon, based on Shapiro–Wilk *p*-values; * indicates statistically significant differences between groups (*p* < 0.05).

**Table 3:** Between-group differences.

Variables	Intervention	Control	f/R^2^	*p*
BS_DIF				
	−2.63 ± −1.05	−0.32 ± −0.04	31.338/0.465	<0.001 *
MD12_DIF				
	−6.37 ± −2.44	0.05 ± 0.21	66.711/0.650	<0.001 *
30STST_DIF				
	1.32 ± 0.14	−0.31 ± 0.72	11.946/0.249	0.001 *
6MWT_DIF				
	112.86 ± −18.23	6.00 ± 1.47	9.279/0.205	0.007 *

DIF: Difference Pre/post-Intervention; BS: Borg Scale; MD12: Multidimensional Dyspnoea-12 questionnaire; 6MWT: Six-Minute Walking Test; 30STST: 30-Second Sit-to-Stand Test; between-group *p*-values come from independent *t*-test/U Mann-Whitney, based on Shapiro-Wilk *p*-values; R^2^: R-Square Effect Size; * indicates statistically significant differences between groups (*p* < 0.05).

## Data Availability

The study protocol and de-identified individual participant data generated during this study are available from the investigators upon reasonable request from the publication. Requests should be directed to the corresponding author by email.

## Supplementary Materials

The following are available online at https://www.mdpi.com/article/10.3390/ijerph18147511/s1, File S1: CONSORT 2010 checklist of information to include when reporting a pilot or feasibility trial.
